# Supplementary material for: Rain and small earthquakes maintain a slow-moving landslide in a persistent critical state
Source: Nat Commun. 2020 Feb 7;11:780. doi: 10.1038/s41467-020-14445-3 (PMC7005893; doi:10.1038/s41467-020-14445-3)
Supplement: Supplementary file 1 — Supplementary Information [file 41467_2020_14445_MOESM1_ESM.pdf]

Supplementary Information for the paper *Rain and small earthquakes maintain a slow-moving landslide in a persistent critical state*.

N. Bontemps et al.

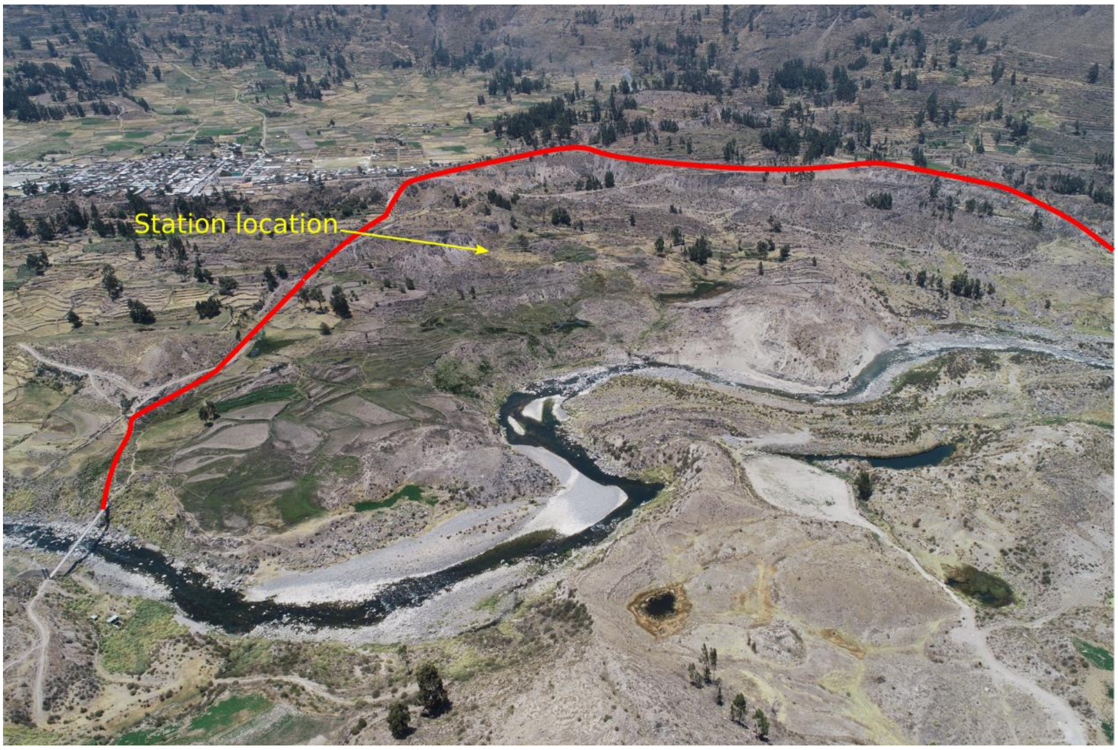

Supplementary Figure 1 | The Maca landslide taken from a drone. The village of Maca is visible on the left, the Colca River is visible at the bottom of the picture. We indicated one of the main scarp in red, defining the fastest part of the landslide together with the location of the hut containing the GPS and the seismic station (orange arrow). More pictures of the landslide can be found in Zerathe et al. (2016)[1]

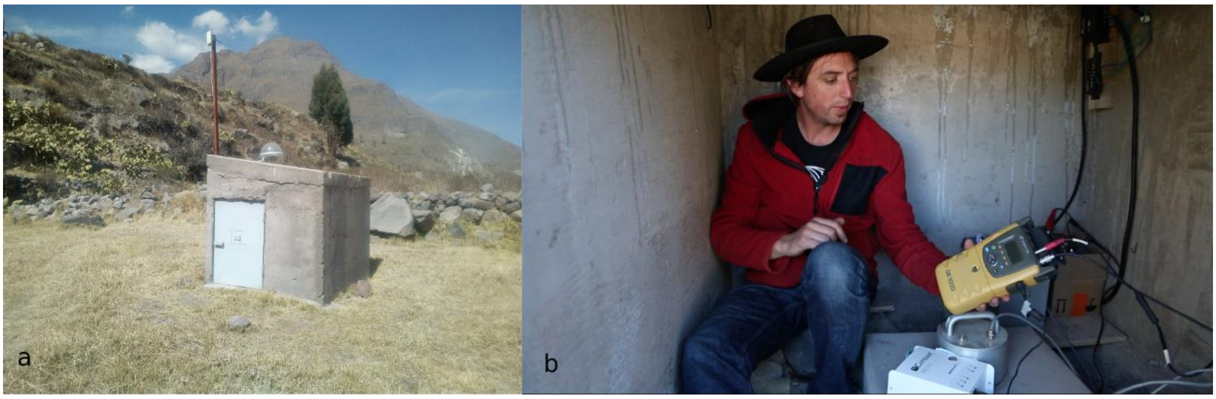

Supplementary Figure 2 | a, Picture of the hut containing the GPS and the seismic station. The antenna of the GPS is visible on the top of the hut. b, interior of the hut, with the GPS and the seismometer installation displayed.

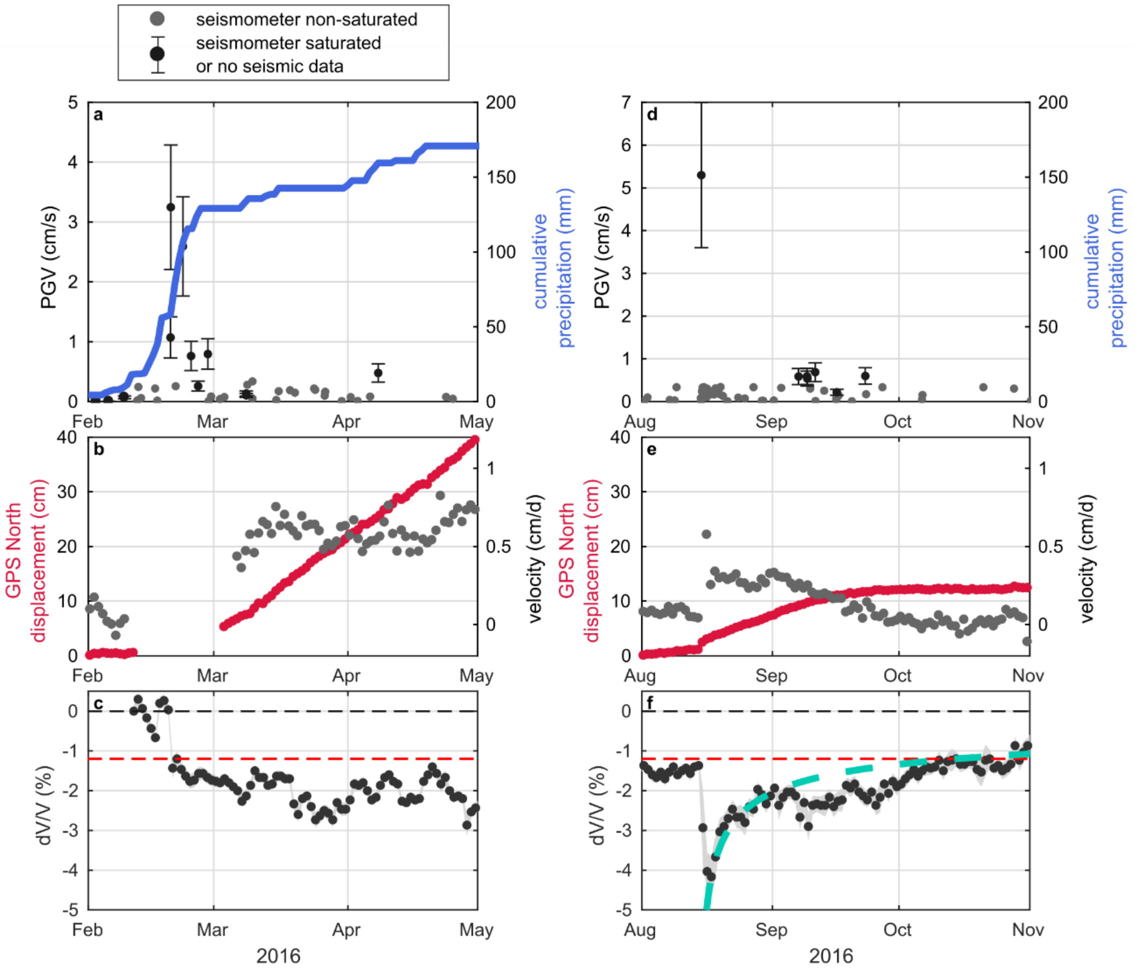

Supplementary Figure 3 | Landslide displacement and dV/V induced by precipitations and/or earthquakes after the two earthquakes in 2016. Close-up for regions in the main text's Figure 2. a) and d) Seismicity reported by the Peruvian Geophysical institute (IGP) for events with a measured or estimated PGV at the landslide site above 0.01cm.s^{-1} (gray dots), and cumulative rainfall over the corresponding rainy season (solid blue line). Earthquakes strong enough to saturate the seismometer located on the landslide and earthquakes for which the seismometer was not working are indicated by black dots. **b) and e)** Time-series for the Maca GPS North cumulative displacement (red) and velocity (gray) with a 1-day sampling rate. **c) and f)** Relative seismic changes to the velocity of the material determined by comparing daily seismic noise correlograms in the 3–8 Hz frequency range (black). The horizontal black dashed line indicates the surface wave velocity on the first day of our study period. The red horizontal dashed line is an empirical threshold used in the text (-1.2%) below which the landslide is in a critical state. The logarithmic curve in f) represents the recovering function proposed by Richter et al., 2014[2,3].

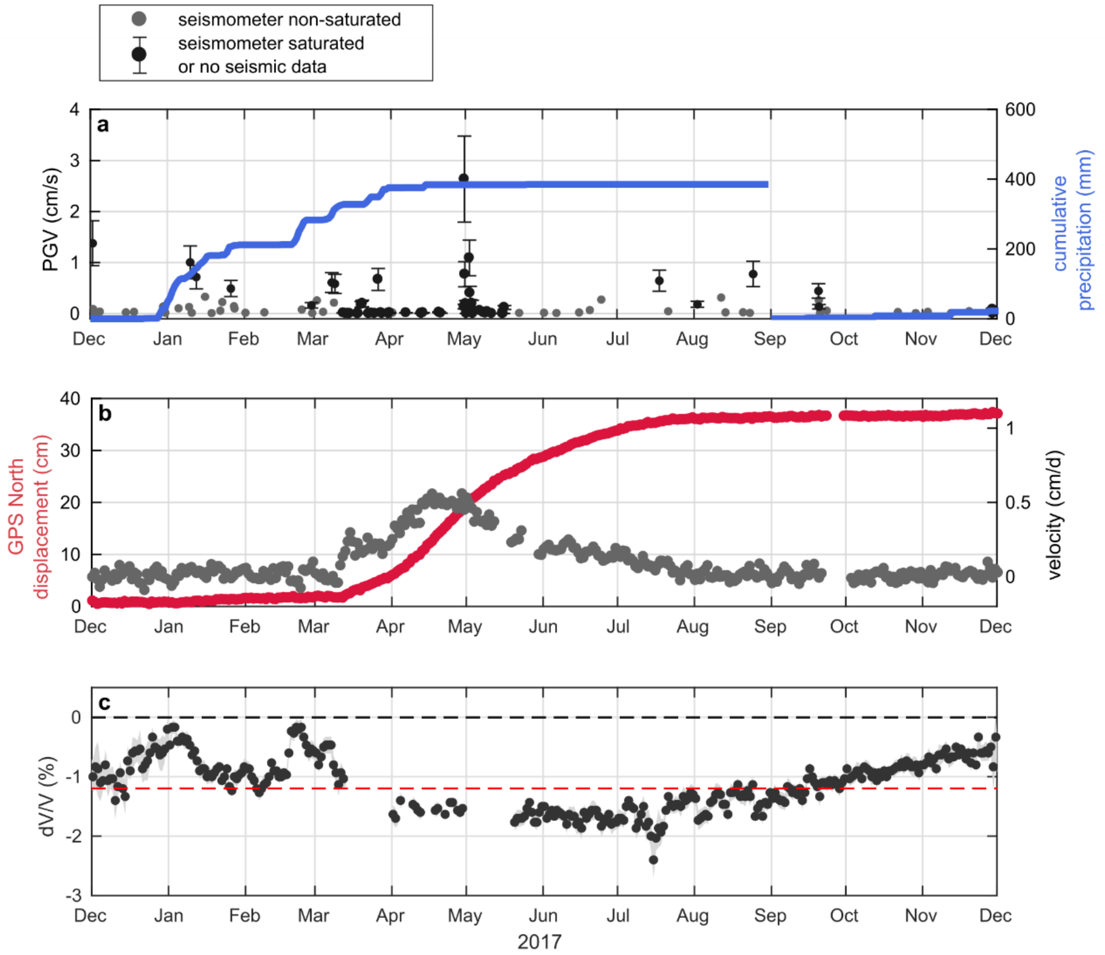

Supplementary Figure 4 | | Landslide displacement and dV/V induced by precipitations and/or earthquakes in 2017. a) Seismicity reported by the Peruvian Geophysical institute (IGP) for events with a measured or estimated PGV at the landslide site above 0.01 cm.s-1 (crosses gray dots), and cumulative rainfall over the corresponding rainy season (solid blue line). Earthquakes strong enough to saturate the seismometer located on the landslide and earthquakes for which the seismometer was not working are indicated by black dots circles. **b)** Time-series for the Maca GPS North cumulative displacement (red) and velocity (gray) with a 1-day sampling rate. **c)** Relative seismic changes to the velocity of the material determined by comparing daily seismic noise correlograms in the 3–8 Hz frequency range (black). The horizontal black dashed line indicates the surface wave velocity on the first day of our study period. The red horizontal dashed line is an empirical threshold used in the text (-1.2%) below which the landslide is in a critical state.

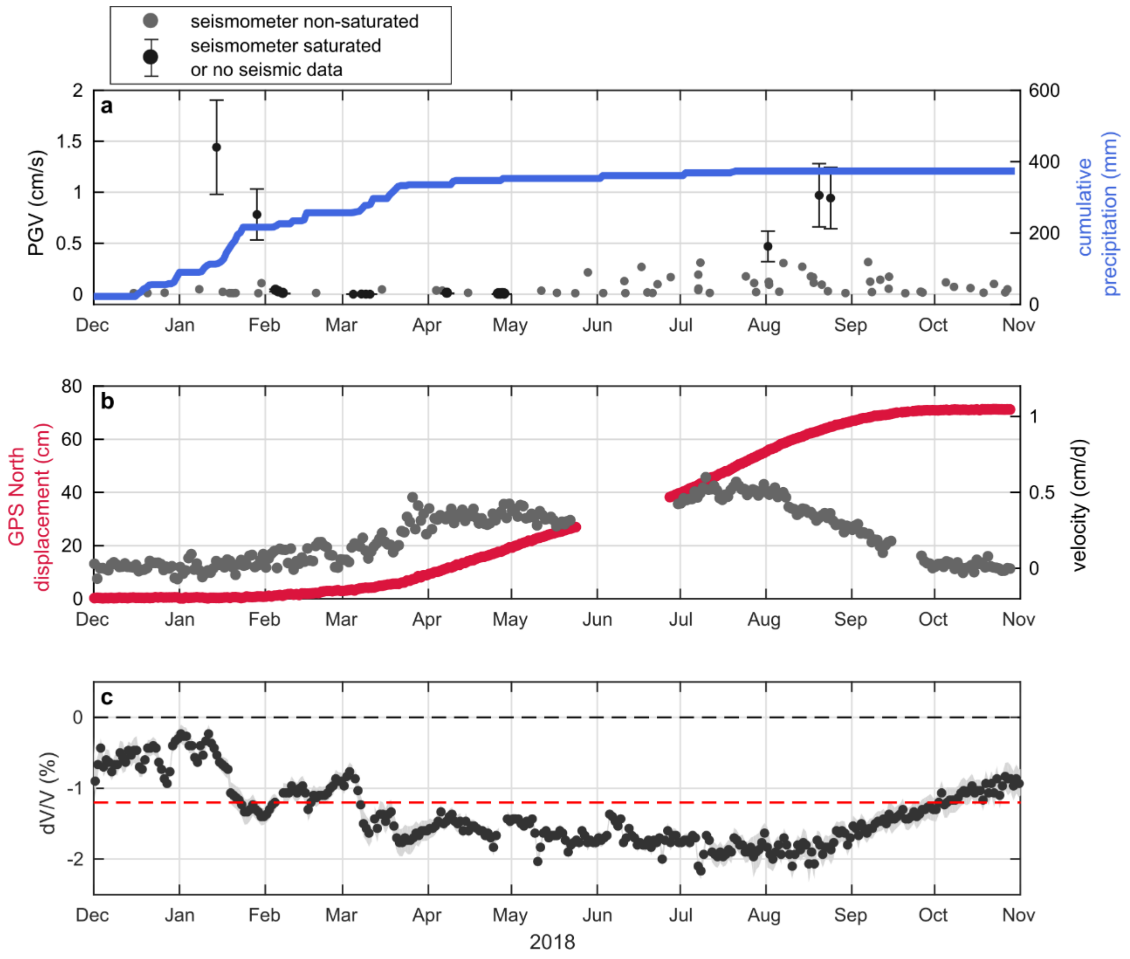

Supplementary Figure 5 | | Landslide displacement and dV/V induced by precipitations and/or earthquakes in 2018. a) Seismicity reported by the Peruvian Geophysical institute (IGP) for events with a measured or estimated PGV at the landslide site above 0.01 cm.s-1 (crosses gray dots), and cumulative rainfall over the corresponding rainy season (solid blue line). Earthquakes strong enough to saturate the seismometer located on the landslide and earthquakes for which the seismometer was not working are indicated by black dots circles. **b)** Time-series for the Maca GPS North cumulative displacement (red) and velocity (gray) with a 1-day sampling rate. **c)** Relative seismic changes to the velocity of the material determined by comparing daily seismic noise correlograms in the 3–8 Hz frequency range (black). The horizontal black dashed line indicates the surface wave velocity on the first day of our study period. The red horizontal dashed line is an empirical threshold used in the text (-1.2%) below which the landslide is in a critical state.

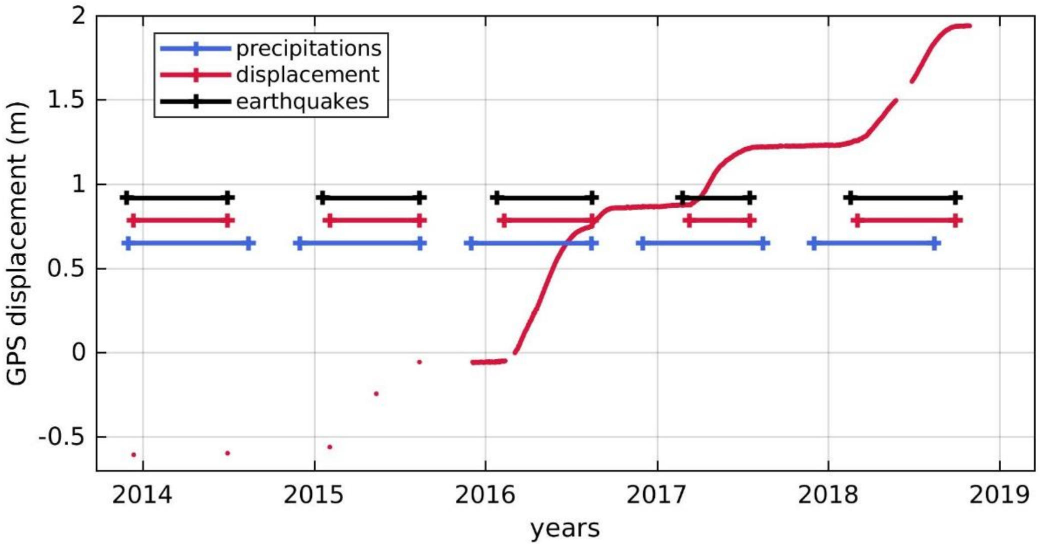

Supplementary Figure 6 | Selected periods for the Figure 3 of the article. GPS measurements of the displacement of the landslide as a function of time from the end of 2013 to 2018. Horizontal bars indicate the periods used in the Figure 3 to calculate the displacement (pink), the cumulative precipitations (blue) and the number of earthquakes (black).

Supplementary Note 1

From Figure 1 of the article, it can be seen that landslide displacements are measured since at least 2003. GPS campaigns were indeed realized since 2001[1] and satellite measurements since 1986 (with much larger uncertainties)[4]. However the measurements were really sparse between 2001 and 2011 (only 3 GPS campaigns and 6 satellite acquisitions), and the satellite acquisitions are also sparse over the whole time period 1986-2014 (16 satellite acquisition), precluding their use for yearly displacement estimates. Frequent GPS campaigns started only in November 2011[1]. Two reasons explain why years 2012 and 2013 are not represented in the Figure 3 of the paper: (1) The year 2012 was an exceptional year in terms of precipitation, where the landslide underwent 7 meters of displacement, i.e., almost seven times its average yearly displacement[1]. Plotting this exceptional year in the Figure 3 would have hidden combined effect of earthquakes and precipitation in more normal rainy seasons. (2) Following the intense activity of the Sabancaya Volcano in 2013, the IGP enlarged his seismometer network in the Colca Valley. The number of earthquakes detected by the IGP, hence, increased after 2013 and the magnitude of the smallest detected events decreased from approximately $Ml = 4$ to around $Ml = 3$. This difference complicates the yearly comparison in terms of the number of earthquakes possibly impacting the landslide before and after 2013.

For all these reasons, Figure 3 is representing only data that we could compare (from 2014 to 2018) (see Supplementary Fig 6 for more information on the Fig 3's creation).

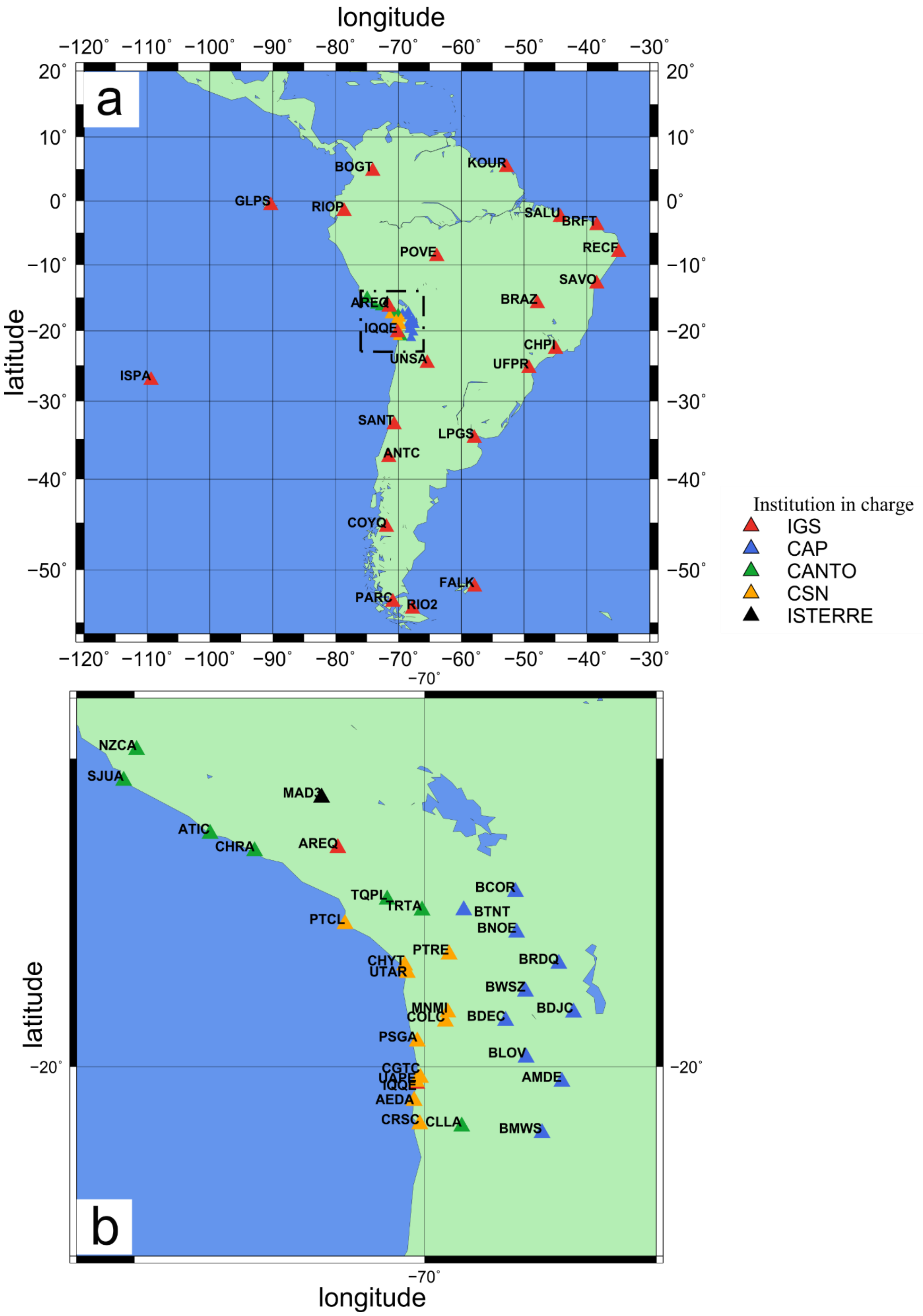

Supplementary Figure 7 | GPS Stations used in this study. a) All the stations used in South America. The square is the area enlarged in **b)**. MAD3 (black triangle) corresponds to the ISTerre GPS station located on the Maca landslide.

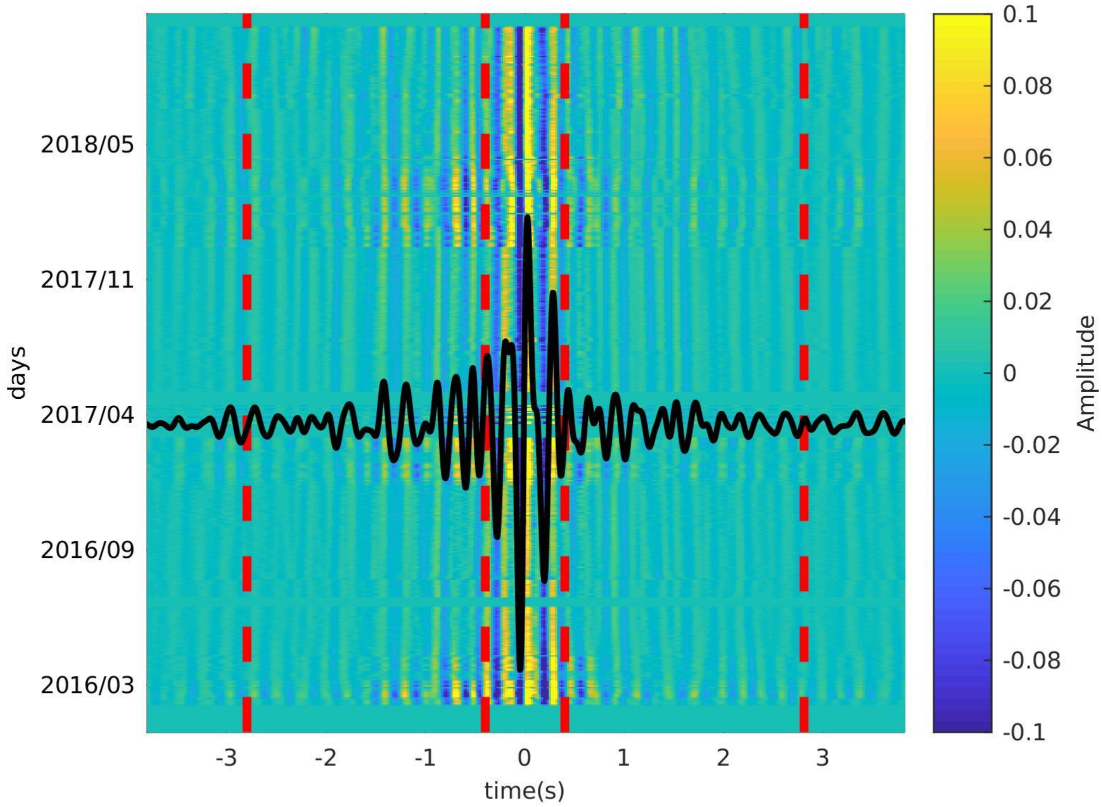

Supplementary Figure 8 | Daily single-station cross-correlation of ambient seismic noise recorded on the Maca landslide. The signal was filtered between 3 and 8 Hz. The reference trace, averaged over all the correlograms, is represented by the continuous black line. Vertical red dotted lines mark the time frame of the coda used to estimate the relative velocity changes dV/V with the stretching method.

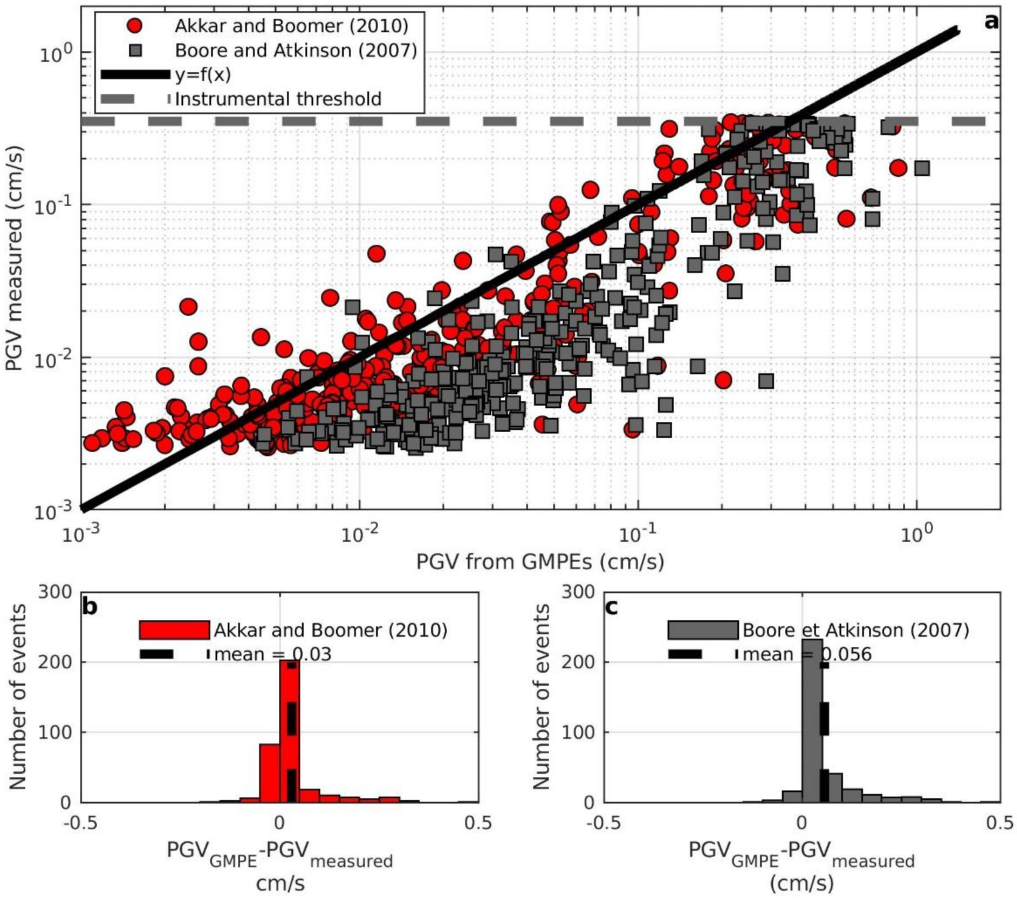

Supplementary Figure 9 | Measured Peak Ground Velocity (PGV) compared to PGV estimated by applying Ground Motion prediction equations (GMPEs). a) Two different GMPE were compared, and the PGV measured was plotted as a function of the PGV estimated for all seismic events with a magnitude greater than 3.5 and a distance of less than 400 km. The normal cutoffs for Boore and Atkinson (2007) is Mw>4 and distance <400 km. For Akkar and boomer (2010) the model is based on earthquakes with magnitude Mw > 5.5 and with a distance <100 km. **b)** and **c)** Histograms showing the differences between measured and estimated PGV determined by applying the Akkar and Boomer(2010) or the Boore and Atkinson (2007) GMPE, respectively.

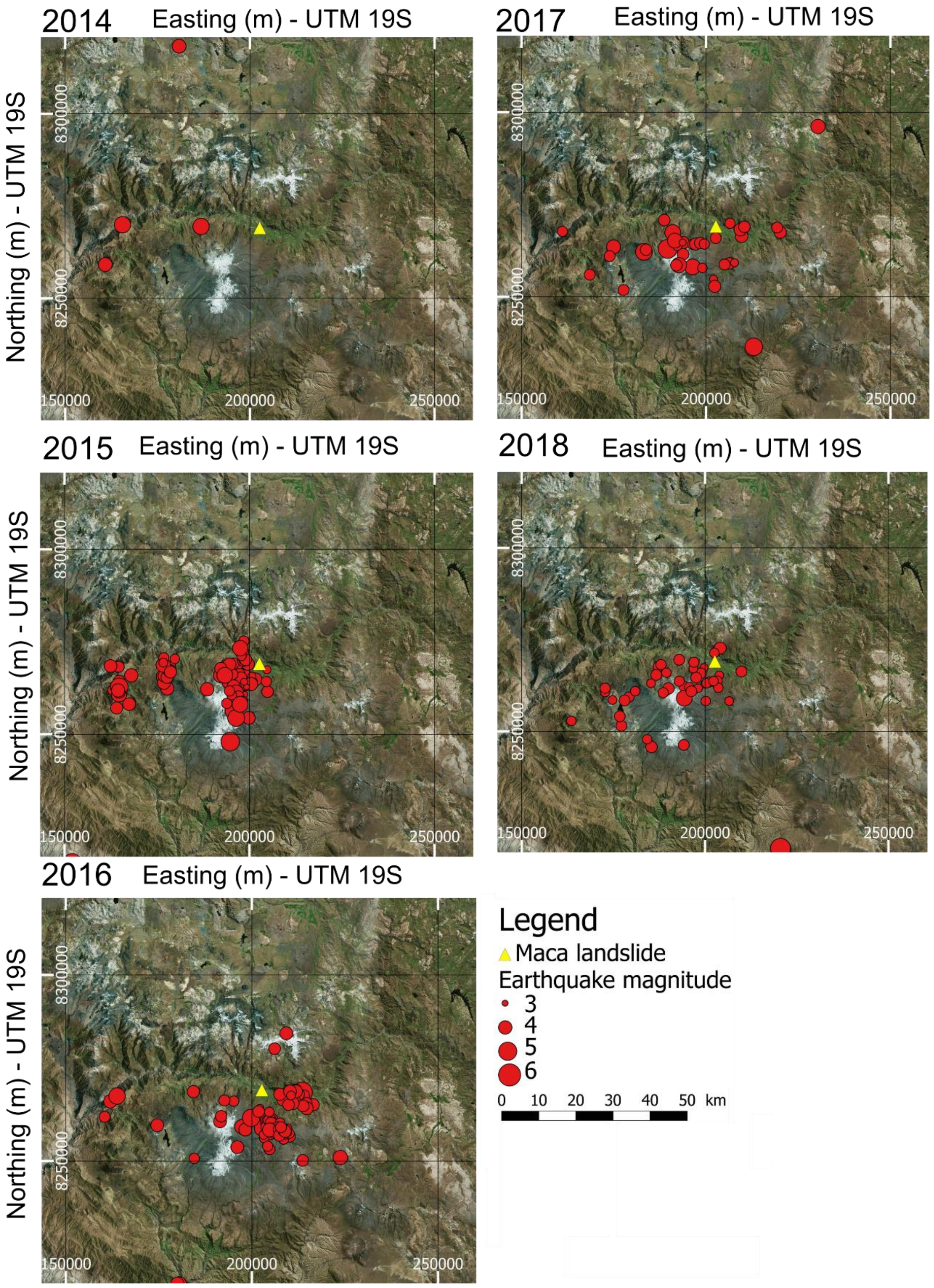

Supplementary Figure 10 | Earthquakes registered by the Peruvian geophysical institute (IGP) between 2014 and 2018. The Maca landslide is represented by the yellow triangle, the maximum distance between the landslide and the earthquake's source (50 km) is delimited by the black circle. The size of the round markers corresponds to the local magnitude of earthquakes.

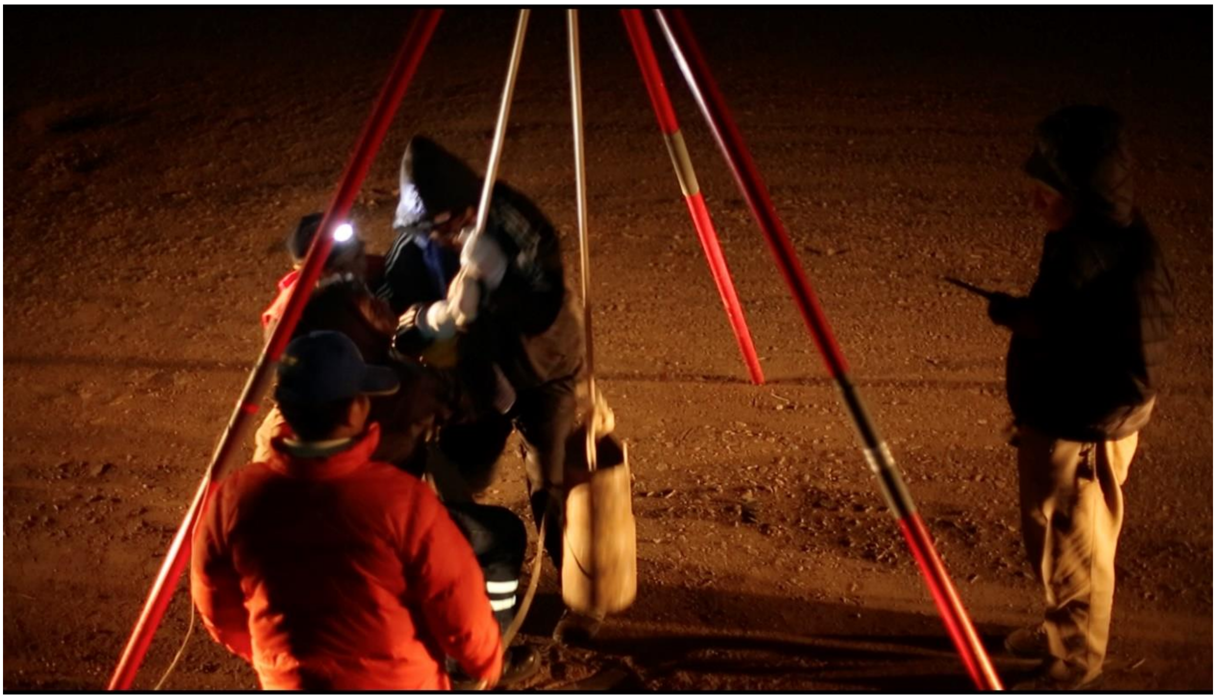

Supplementary Figure 11 | Estimation of the P wave velocities in the Maca landslide. Picture of the source system. An 80-kg mass was pulled up to approximately 2 m high using a tripod and a pulley and dropped onto a metallic plate. Data were acquired at night to diminish the impact of wind and anthropogenic noise (mainly coming from a road attracting considerable tourist traffic) on the signal recorded.

Supplementary Note 2 : Geophysical investigation

Active seismic acquisitions on the Maca landslide were used in April 2016 to estimate the velocity of the P- and S-waves. Data were acquired at night to limit the impact of wind and anthropogenic noise. The source was an 80 kg mass pulled up to 2 m height using a system consisting of a pulley and a tripod (see Supplementary Fig. 6). The velocity of the P-waves was extracted by applying the refraction method. The results show a first shallow layer a few meters thick, with P-wave velocities of ≈ 230 m/s. This layer corresponds to an old debris avalanche which was described in a previous study[1]. Below this shallow layer, the P-waves have velocities of ≈ 1900 m/s, indicating a completely saturated lacustrine layer, assumption based on the fact that the acquisition was realized at the end of the rainy season. These P-waves were later used to better determine the limitations of the model inferred from the dispersion of the surface waves and to retrieve the velocity of the shear waves at depth.

The surface wave inversion technique was applied to generate a model of the S-wave velocity at depth in the landslide. The Rayleigh wave phase velocity dispersion curve was picked using Geopsy software (http://www.geopsy.org) (Supplementary Fig. 12a). The dispersion curve obtained was used to extrapolate the speed of the wave over a frequency range from 4 Hz to 9 Hz. Dispersion curves were inverted in Geopsy software

by applying the enhanced neighborhood algorithm[5] (Supplementary Fig. 12b). For all inversions, the corresponding models of S-wave velocities are represented in Supplementary Figure 12c. Several models have good misfit values, below 3%. From these models the velocity of the S-waves was estimated to be near 110 m/s in the first layer of rock avalanche and to travel at a speed of 350 m/s in the layer composed of lacustrine deposits. The limit between the two different layers changed laterally between 5 and 10 meters depending on the position of the array on the landslide.

Active seismic acquisitions only correspond to snapshots of the velocity of P and S waves at a given time. Given that these acquisitions were realized at the very end of the rainy season, we assume, for simplification, that P and S wave velocities of the second layer correspond to a completely saturated lacustrine deposit layer. This assumption, then allow us to calibrate the parameters in Biot-Gassmann equations (see method section) to retrieve the velocity of P and S waves as a function of the saturation.

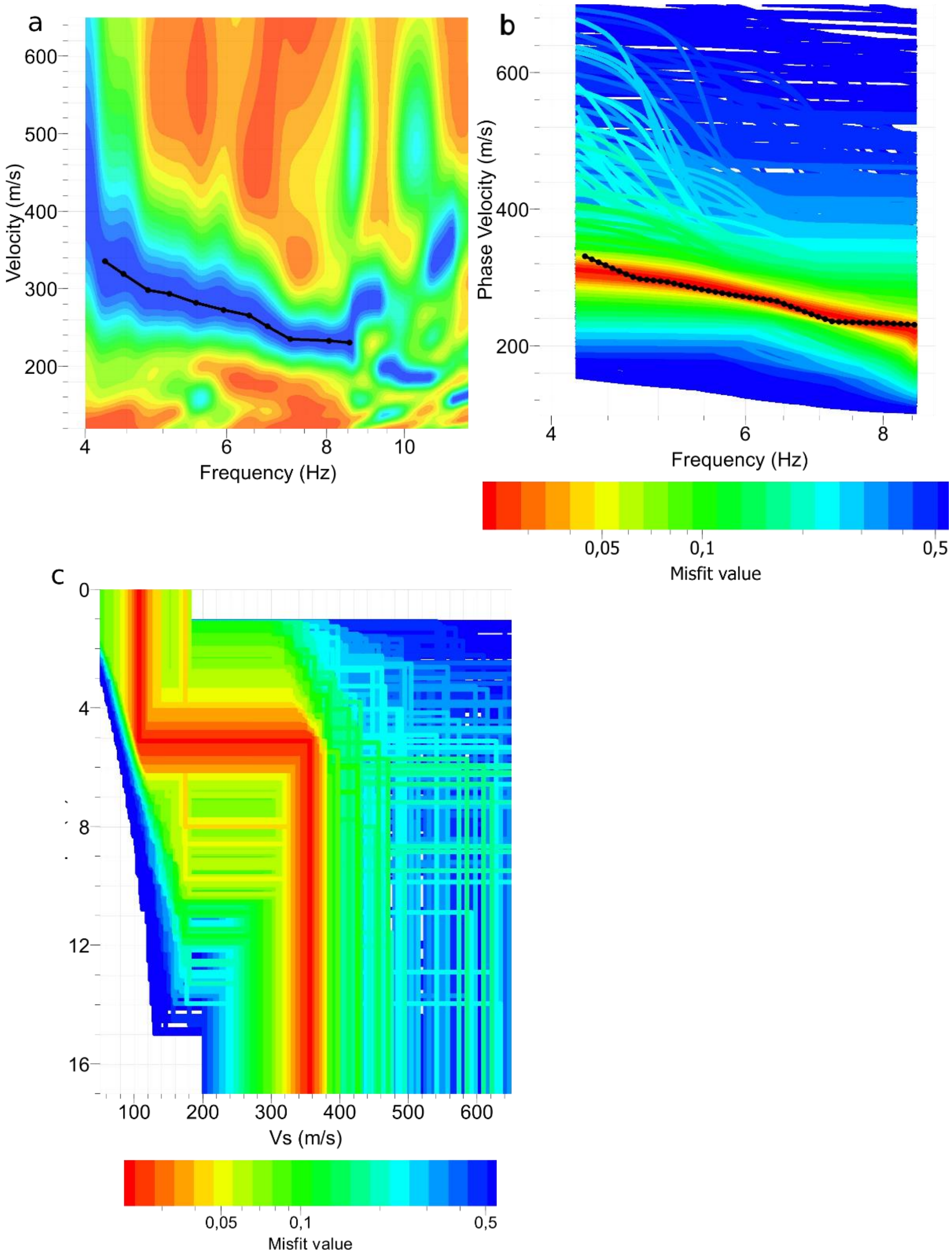

Supplementary Figure 12 | Surface wave dispersion technique applied in Maca. a) Phase velocity dispersion curve obtained from signals recorded on geophones aligned with an inter-trace distance of 10 m from the upper part of the landslide toward its toe. The source was located at 70 m from the first geophone. **b)** Best fitting dispersion curves corresponding to the Vs models given in **c)** and to the velocity dispersion curve selected in **a)**.

Supplementary Note 3: Depth-sensitivity of the surface waves as a function of the frequency range

The depth-sensitivity of the Rayleigh waves was examined as a function of the frequency band thanks to the *gpdc* package available in the Geopsy software. This package provides the dispersion curves of the Rayleigh waves as a function of frequency by giving a model of the soil as an input. The dv/v generated by different perturbations of the S-wave velocity in the medium can then be compared at different depths. This was done for a perturbation of the medium of 14% over a depth of 50 cm, varying between 5 and 50 m. The data shown in Supplementary Figure 13a indicate that surface waves between 3 and 8 Hz are mainly sensitive to soil damage occurring between 5 and 40 m. This result is corroborated by the fact that no dv/v variations were observed after the two largest earthquakes at frequencies lower than 2 Hz (Supplementary Fig. 14).

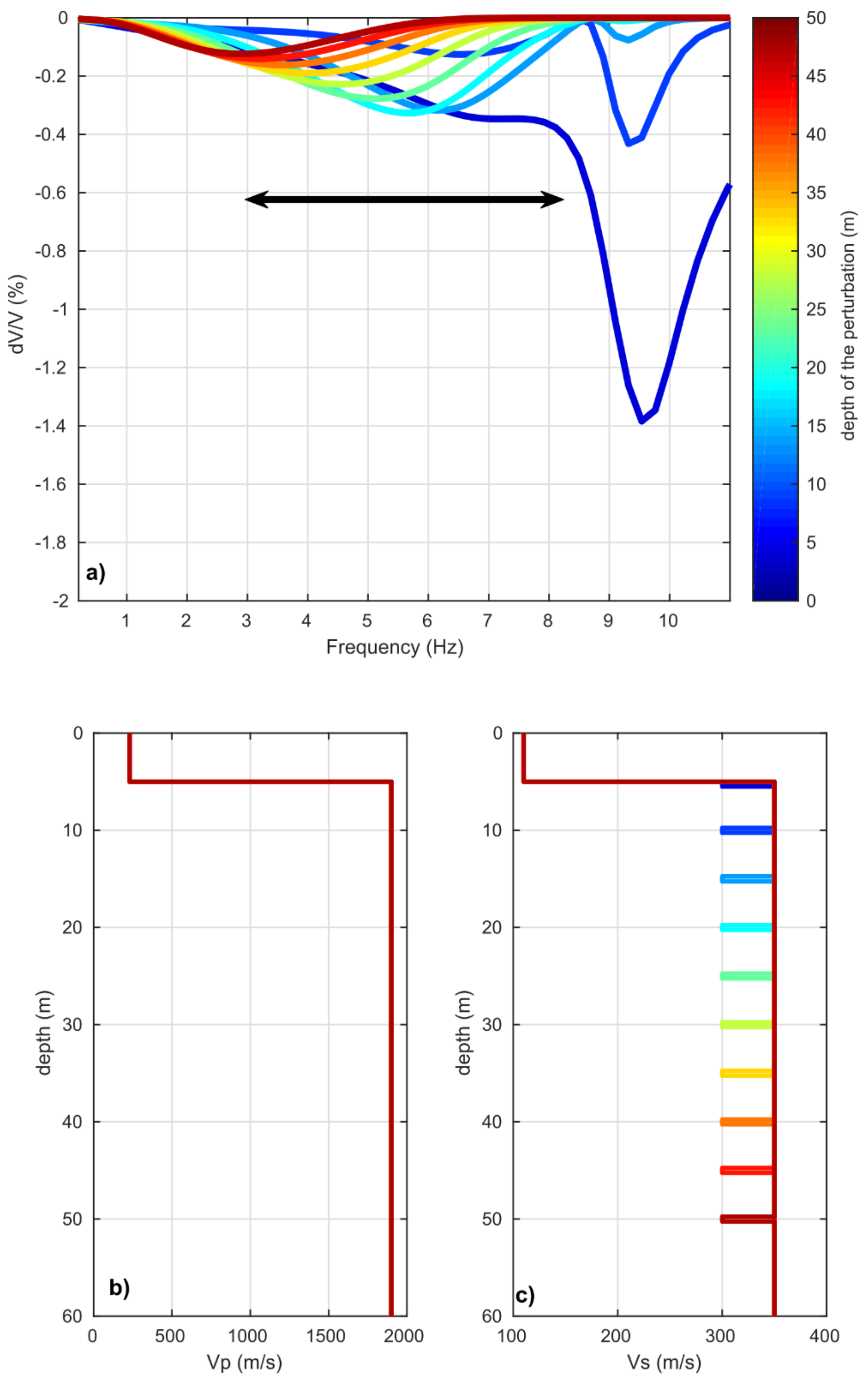

Supplementary Figure 13 | a, dv/v expected to be observed by the seismometer as a function of the frequency for the perturbations displayed in **b** and **c**. **b** and **c,** velocity models of the soil as a function of depth for the P- and S-waves respectively, with a perturbation of 14% of the S-wave velocity over 50 cm varying between 5 and 50 m depth. The perturbation at 5 m depth was only 3%. The arrow represents the mains studied frequency band.

Supplementary Note 4: Liquefaction model

In order to model the impact of the liquefaction of the sliding surface (undrained cyclic loading), we first used a velocity model with two layers, one constituted of avalanche deposits (Vp= 230 m/s and Vs = 110 m/s) and one of lacustrine deposits, saturated at 25% (see method section for the computation of Vp and Vs as a function of the saturation). We obtained Vp = 900 m/s and Vs=375 m/s for the second layer. Then, for undrained loading to occur, the sliding surface must be in a water table, or in a fully saturated soil. Hence we modeled a 5-m fully saturated layer above the sliding surface (hence between 35 and 40 meters depth) with P and S wave velocities of 1900 m/s and 350 m/s, respectively. This constitutes the model prior to the liquefaction (see Supplementary Figure 14a).

To imitate the impact of undrained loading, we decreased Vs by 45% based on observations and models[6] when the sliding surface of the Pont Bourquin landslide fluidized. This model considers that the sliding surface fluidizing had a thickness of 2 meters. A shearing plane's thickness of one to two meters is common for slow-moving landslide[7–9] and hence modeled the decrease of velocity over two meters shearing plan at 40 meter depth (see Supplementary Figure 14a) . We chose not to change Vp and the density of the soil during undrained loading, as they should not be that much influenced by the liquefaction.

The black curve in Supplementary Figure 14 b indicates the trend the dv/v should take as a function of the frequency in case of undrained loading as modeled previously. This degree of perturbations in the medium would generate a decrease of at least 0.75% of the dV/V over the 0.2 Hz to 2 Hz frequency band and of 1.4% over the 3 Hz to 8 Hz frequency band ($\int_{f1}^{f2}(dv/v)df/(f2-f1)$). In addition, due to the main variation occurring around 40 m, one should observe a peak of dv/v variations around 3 Hz. The sliding surface being at least below 40 meters, this peak can only be at or lower than 3 Hz. In Supplementary Figure 14b, we plotted the dv/v measured for different frequency bands (the squares representing the middle of the studied frequency band). Overall, we can say that the measured dv/v drops are way too low below 5 Hz and too high above 5 Hz to be explained by undrained loading.

The model tells us that, if undrained loading occurs at lower depth (e.g., 20 meters), the expected magnitude of the dv/v drop together with the impacted frequencies will be higher. This would be even more in contradiction to our measurements and hence, the existence of a layer impacted by undrained loading at lower depth seems not in agreement with our observations.

We therefore conclude that undrained loading is not the mechanism causing displacement of the landslide in August. The same is applicable in February.

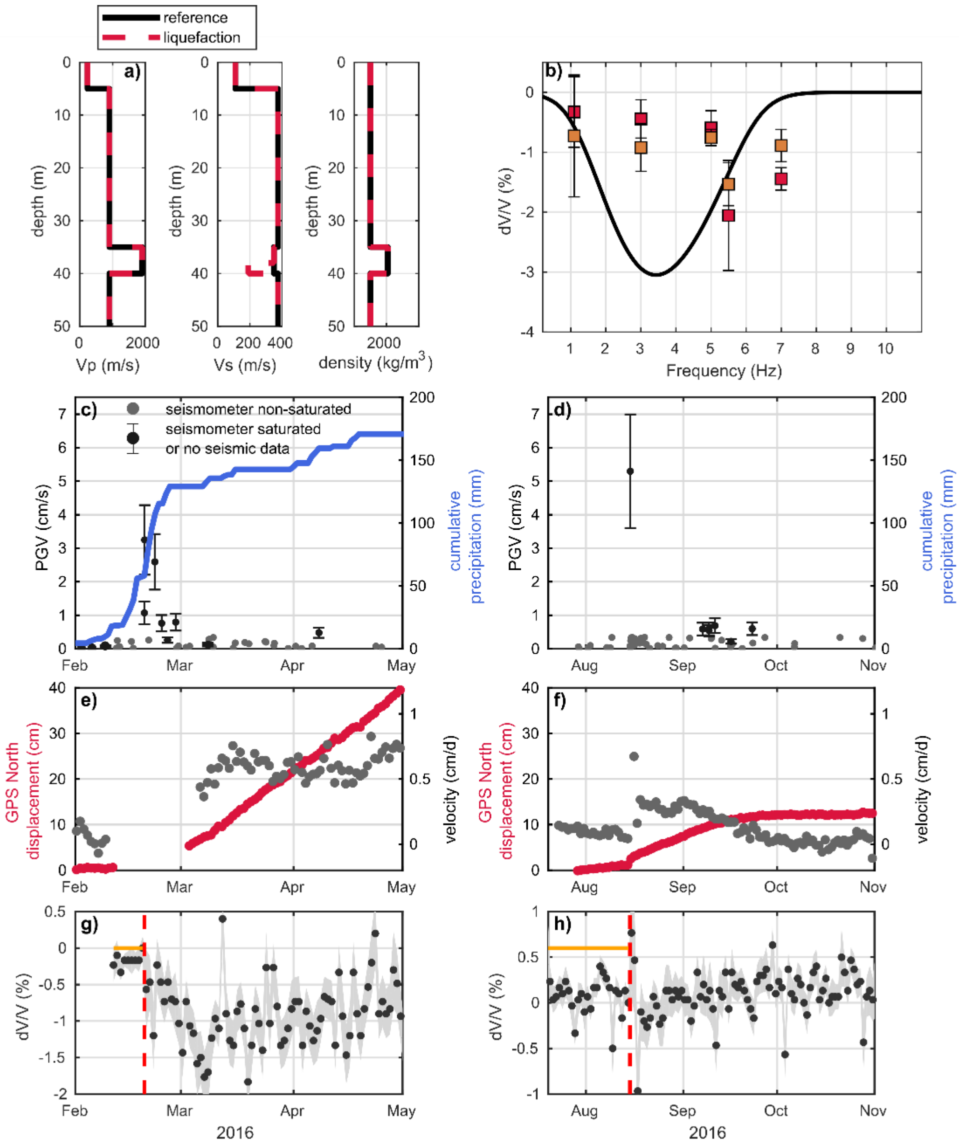

Supplementary Figure 14 | a) velocity models of the soil as a function of depth for the P- and S-waves together with the density, modeling the liquefaction of a 2 m layer representing the sliding surface , at 40 m depth. **b** dv/v expected to be observed by the seismometer as a function of frequency for the perturbation displayed in **a.**Times series of the dv/v were first calculated in the different frequency bands (0.2-2Hz, 2-4 Hz, 4-6 Hz, 6-8 Hz and 3-8 Hz). Chosen parameters are frequency band dependent and are presented in the method section. The drop of dv/v for the two large events (ML> 5) is calculated by subtracting the average of the dv/v of the three days following each earthquake to the three days preceding them. **c)** and **f)** Seismicity reported by the Peruvian Geophysical Institute (IGP) for events with a measured or estimated PGV at the landslide site above 0.01cm.s^{-1} (gray dots), and cumulative rainfall over the corresponding rainy season (solid blue line). Earthquakes strong enough to saturate the seismometer located on the landslide and earthquakes for which the seismometer was not working are indicated by black dots. **d)** and **g)** Time-series for the Maca GPS North cumulative displacement (red) and velocity (gray) with a 1-day sampling rate. **e)** and **h)** dv/v observed in the 0.2 Hz to 2 Hz frequency

range. The references used for the stretching step correspond, here, to the orange horizontal lines. They were chosen shorter here in order to diminish the signal-to-noise ratio.

Supplementary References

1. Zerathe, S. *et al.* Morphology, structure and kinematics of a rainfall controlled slow-moving Andean landslide, Peru: The Maca slow-moving Andean landslide. *Earth Surface Processes and Landforms* **41**, 1477–1493 (2016).
2. Richter, T., Sens-Schönfelder, C., Kind, R. & Asch, G. Comprehensive observation and modeling of earthquake and temperature-related seismic velocity changes in northern Chile with passive image interferometry. *Journal of Geophysical Research: Solid Earth* **119**, 4747–4765 (2014).
3. TenCate, J. A. Slow Dynamics of Earth Materials: An Experimental Overview. *Pure Appl. Geophys.* **168**, 2211–2219 (2011).
4. Bontemps, N., Lacroix, P. & Doin, M.-P. Inversion of deformation fields time-series from optical images, and application to the long term kinematics of slow-moving landslides in Peru. *Remote Sensing of Environment* **210**, 144–158 (2018).
5. Wathelet, M. An improved neighborhood algorithm: Parameter conditions and dynamic scaling. *Geophysical Research Letters* **35**, n/a–n/a (2008).
6. Mainsant, G. *et al.* Ambient seismic noise monitoring of a clay landslide: Toward failure prediction: seismic noise monitoring of a landslide. *Journal of Geophysical Research: Earth Surface* **117**, (2012).
7. Jongmans, D., Bièvre, G., Schwartz, S., Renalier, F. & Beaurez, N. Geophysical investigation of the large Avignonet landslide in glaciolacustrine clays in the Trièves area (French Alps). *Engineering Geology* **109**, 45–56 (2009).
8. Kane, W. F., Beck, T. J. & Hughes, J. J. Applications of time domain reflectometry to landslide and slope monitoring. in *Second International Symposium and Workshop on Time Domain Reflectometry for Innovative Geotechnical Applications* 305–314 (Infrastructure Technology Institute at Northwestern University Evanston, IL, 2001).
9. Simeoni, L. & Mongiovì, L. Inclinometer monitoring of the Castelrotto landslide in Italy. *Journal of geotechnical and geoenvironmental engineering* **133**, 653–666 (2007).
10. Akkar, S. & Bommer, J. J. Empirical Equations for the Prediction of PGA, PGV, and Spectral Accelerations in Europe, the Mediterranean Region, and the Middle East. *Seismological Research Letters* **81**, 195–206 (2010).
11. Boore, D. M. & Atkinson, G. M. Boore-Atkinson NGA Ground Motion Relations for the Geometric Mean Horizontal Component of Peak and Spectral Ground Motion Parameters. *PEER Report 2007/01* 110 (2007).
